# Determination of MDPBP in postmortem blood samples by gas chromatography coupled with mass spectrometry

**DOI:** 10.1007/s00706-016-1780-0

**Published:** 2016-06-15

**Authors:** Marek Wiergowski, Mateusz K. Woźniak, Marzena Kata, Marek Biziuk

**Affiliations:** 1 Chair and Department of Forensic Medicine, Faculty of Medicine, Medical University of Gdańsk, Gdańsk, Poland; 2 Department of Analytical Chemistry, Faculty of Chemistry, Gdańsk University of Technology, Gdańsk, Poland

**Keywords:** Cathinones, Drug research, Extraction, Gas chromatography, Toxicological screening

## Abstract

**Abstract:**

MDPBP (1-(3,4-methylenedioxyphenyl)-2-(1-pyrrolidinyl)-1-butanone) is a new psychoactive substance sold on the black market. It has been a controlled drug of abuse in Poland and China since 2015 as some toxic and fatal cases connected with use of synthetic cathinone derivatives were observed. The fatal case outlined here concerns a 19-year-old man, who was found dead with an envelope containing white powder lying nearby the cadaver. The analyses of the powder revealed a presence of MDPBP. Due to this, blood was tested for routine toxicological analysis for traditional drugs and for MDPBP by liquid–liquid extraction procedure with 1-chlorobutane followed by GC–MS analysis. Full validation of proposed method was performed. Limit of detection and limit of quantification were 10.1 and 30.4 ng/cm^3^, respectively. Calibration curve was linear in studied concentration range (25–1000 ng/cm^3^) with a correlation coefficient 0.9946. The trueness and inter-day precision expressed as recoveries and CV values were investigated at 3 concentrations: 25, 250, and 1000 ng/cm^3^. The CV values were less than 20 % in the lowest concentration and less than 15 % in other concentrations what met the internationally established acceptance criteria for bioanalytical methods. It indicates good precision and accuracy of the method. The analysis of blood sample showed very high concentration of MDPBP (9.32 µg/cm^3^), which suggests possibility of overdosing. To the best of our knowledge, this is the first work which presents determination of MDPBP in blood by GC-EI-MS method and the third fatal accident report of MDPBP abuse.

**Graphical abstract:**

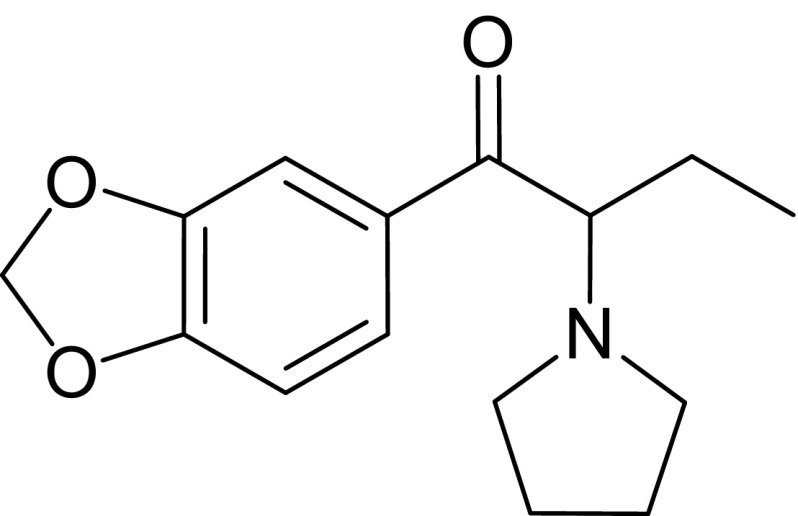

## Introduction

The interest in synthetic cathinone derivatives is a reflection of their diverse range of biologically active properties. Synthetic cathinones can stimulate central nervous system. Therefore, medicinal exploration of cathinone derivatives is not surprising. One of the pharmacological properties of these compounds is the inhibition of monoamine uptake transporters. It makes them an interesting target for therapeutic applications, e.g., antidepressant therapy, neurodegeneration, drug addiction, and smoking cessation. However, a number of cathinone derivatives offer also psychostimulatory and entactogenic properties and are extensively available on black market as new psychoactive substances (NPS) under the names: “legal highs”, “designer drugs”, “research chemicals”, “bath salts”, or “plant food”. Synthetic cathinones represent approximately two thirds of the NPS available in the new drug market [1]. Recently, there has been a significant rise in the popularity of these compounds especially among young people on account of their stimulant properties and due to the fact that they are perceived to be pure and safe [2]. A novel group of these drugs as beta-ketone amphetamine analogs contain α-pyrrolidinophenone unit originated in China and, to a lesser extent, in India and in Europe [3–5]. Structures of some drugs contain α-pyrrolidinophenone unit are presented in Scheme 1 and Table 1.

**Figure:**
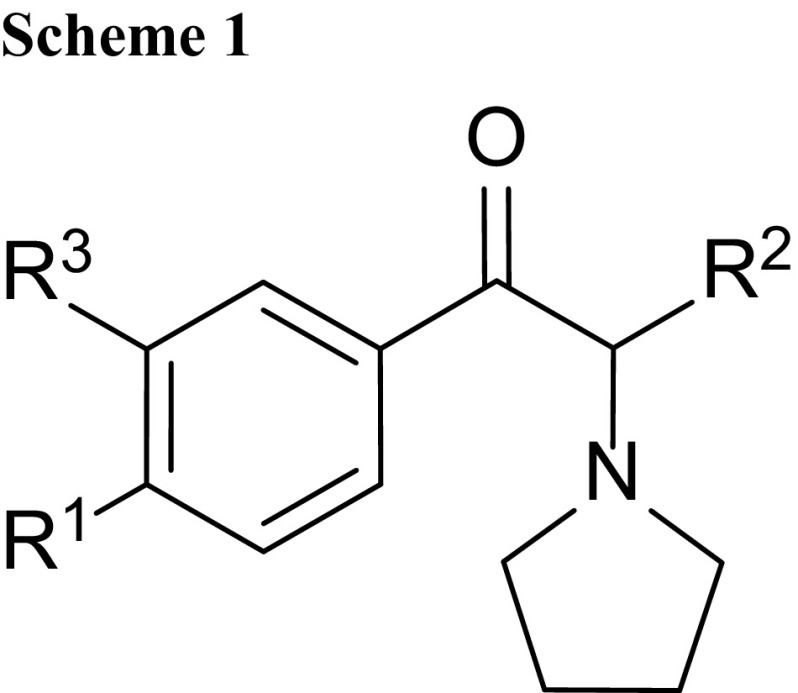

**Table 1:** Structures of some drugs contain α-pyrrolidinophenone unit [4]

Name (CAS number; IUPAC name)	R^1^	R^2^	R^3^
Alpha-PPP (19134-50-0; (RS)-1-phenyl-2-(1-pyrrolidinyl)-1-propanone)	H	CH_3_	H
MPPP (1313393-58-6; (RS)-1-(4-methylphenyl)-2-(1-pyrrolidinyl)-1-propanone)	CH_3_	CH_3_	H
Pyrovalerone (3563-49-3; (RS)-1-(4-methylphenyl)-2-(1-pyrrolidinyl)-pentan-1-one)	CH_3_	C_3_H_7_	H
MPBP (1214-15-9; (RS)-1-(4-methylphenyl)-2-(1-pyrrolidinyl)-1-butanone)	CH_3_	C_2_H_5_	H
Alpha-PVP (14530-33-7; (RS)-1-phenyl-2-(1-pyrrolidinyl)-1-pentanone	H	C_3_H_7_	H
MDPPP (24698-57-5; (RS)-1-(3,4-methylenedioxyphenyl)-2-(1-pyrrolidinyl)-1-propanone	**–**O**–**CH_2_ **–**O**–** (R^1^ **–**R^3^)	CH_3_	**–**
MDPBP (24622-60-4; (RS)-1-(3,4-methylenedioxyphenyl)-2-(1-pyrrolidinyl)-1-butanone)	–O–CH_2_–O– (R^1^–R^3^)	C_2_H_5_	–
MDPV (687603-66-3; (RS)-1-(benzo[d][1,3]dioxol-5-yl)-2-(pyrrolidin-1-yl)pentan-1-one)	**–**O**–**CH_2_ **–**O**–** (R^1^ **–**R^3^)	C_3_H_7_	–

MDPBP (3′,4′-methylenedioxy-α-pyrrolidinobutyrophenone) is a psychoactive compound developed in the 1960s. It is available for sale since 2009 (e.g., via the internet or in black market shops) as one of the novel designer drugs. It is usually mixed with flephedrone, pentedrone, pentylone, MPPP, and MDPV. Its presence was confirmed in the seized “legal highs”, such as: White Fiz, Vanilla Sky, and Bath Salts. Psychoactive dose of MDPBP which affects central nervous system, ranges from 50 to 100 mg. It is also usually standard dose which is taken by abusers. MDPBP is the most commonly administered via oral ingestion, nasal insufflations, smoking, or intravenous injections. The pharmacokinetics and psychoactive effects of this drug have not been fully researched yet, but according to testimony of its users, they are similar to ephedrine, amphetamine, cocaine and to other substances containing α-pyrrolidinophenone unit. Its use has been reported to cause states of euphoria, agitation, hallucinations, and aggressive behavior. Overdoses cause confusion, acute poisoning, increase heart rate, high blood pressure and finally may lead to arrhythmia, myocardial ischemia, and death [5–7].

The case described herein concerns a 19-year-old man who was found dead and naked on the field. Small bag with white powder was also found nearby. It was believed, that the powder had been taken by the man before death. However, his friends testified that the deceased was on the party the day before accident, but he unexpectedly fled. They also reported that man had drunk only approximately 100 cm^3^ of vodka and the drugs were not used during the party. The victim was not agitated or aggressive. Moreover, according to his father declarations the young man did not have a habit of taking drugs or high doses of alcohol.

Analysis of the powder by mass spectrometry methods revealed the presence of MDPBP as the only psychoactive compound in the powder. Due to necessity of confirmation of MDPBP intake by victim before death, the aim of this study was to identify and determinate the concentration of MDPBP in blood obtained during autopsy. However, abusers often take more than one drug. Therefore, blood sample was also investigated for psychoactive classical drugs: amphetamine, methamphetamine and their derivatives, cannabinoids, opiates, benzodiazepines, cocaine and their metabolites by ELISA immunoenzymatic test (Neogen) as it is performed during routine toxicology analysis. The screening test was positive for cannabinoids. Therefore, the identification and quantification of Δ^9^-tetrahydrocannabinol (Δ^9^-THC) and its main metabolite 11-nor-Δ^9^-tetrahydrocanabinol-9-carboxylic acid (THC-COOH) by derivatization and gas chromatography negative ion chemical ionization mass spectrometry (GC-NCI-MS) and electron impact ionization (GC-EI-MS) analysis, respectively, according to developed method by Kała and Kochanowski, was also performed [8].

## Results and discussion

### Method validation

**Fig. 1:**
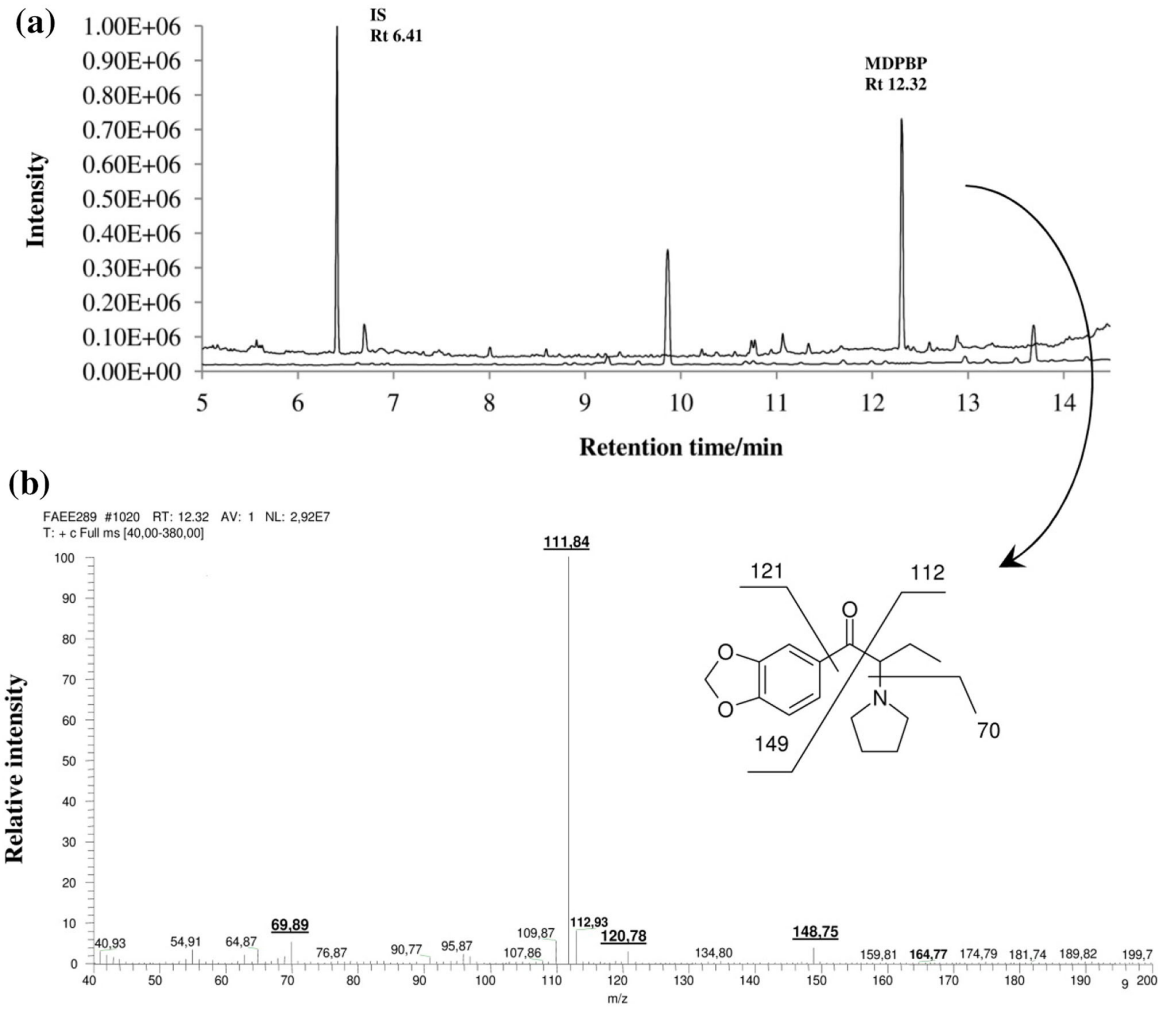
**a** GC-EI-MS-SCAN chromatogram for mixture of IS solution with standard solution of MDPBP (500 ng/cm^3^) in methanol (*upper* chromatogram) and chromatogram obtained by GC-EI-MS-SIM for the extracted blank blood sample used to validation procedure (*lower* chromatogram); **b** EI-MS (70 eV) proposed mass spectral fragmentation pattern of MDPBP

Full validation of the method was performed. No MDPBP, rac-methamphetamine-D_5_ (mAMP-D_5_) as an internal standard (IS), and additional peaks due to endogenous substances that could have interfered were reported in blank samples and in samples investigated for selectivity at the retention times of the analytes (Fig. 1). No carry-over effect was observed. Therefore, investigated method is characterized by high specificity and can be applied for determination of MDPBP in blood. The data of calibration curves to establish matrix effect (*ME*) were summarized in Table 2.

**Table 2:** Calibration curve’s equations for standard solutions and blood’s extracts spiked with analytes in the concentration range 25–1000 ng/cm^3^

Matrix	Calibration curves		Slope of matrix/standard solution	*ME*/%
	Regression equation	*R* ^2^		
Methanol	*y* = 0.0013*x* − 0.0041	0.9904	0.692	69.2
Blood’s extracts	*y* = 0.0009*x* + 0.025	0.9965		

The value of *ME* below 100 % indicates suppression of signal by co-extracted compounds. In view of significant matrix effect, calibration curve prepared in blood instead of external calibration is recommended. To compensate the variability of the detector responses during analysis and losses of analytes in the extraction and sample preparation steps (correction of recovery), the internal standard calibration was performed. The calibration curve demonstrated a linear fit with a correlation coefficient 0.9946 in the studied concentration range (25–1000 ng/cm^3^). The limit of detection (LOD) and limit of quantification (LOQ) values were 10.1 and 30.4 ng/cm^3^ for weighted linear calibration curve. The following regression parameters were obtained: *a* = 0.00099; *b* = 0.031; *S*_*a*_ = 0.000019; *S*_*b*_ = 0.0034. The values of recoveries and CV for trueness and inter-day precision were summarized in Table 3.

**Table 3:** Trueness and inter-day precision of assay

Recovery (CV)/% (*n* = 6)				
Concentration/ng cm^−3^	Trueness			Inter-day precision
	Day 1	Day 2	Day 3	
25	99.4 (9.2)	103.0 (4.2)	102.7 (9.5)	101.8 (7.5)
250	109.8 (9.2)	107.2 (1.1)	105.9 (2.0)	107.7 (5.5)
1000	92.4 (5.1)	94.7 (4.1)	92.1 (1.9)	93.1 (3.9)

The recovery data and CV values showed that proposed method is characterized by high accuracy and precision. Moreover, the mean values of CV are less than 20 % in the lowest concentration and less than 15 % in other concentrations what met the internationally established acceptance criteria for bioanalytical methods [9, 10].

### Analysis of real samples

The chemical and toxicological study of postmortem blood samples revealed concentration of MDPBP—9.32 µg/cm^3^. Additionally, in blood samples psychoactive Δ^9^-THC was detected below LOQ level (<0.001 µg/cm^3^) and its inactive main metabolite THC-COOH (0.006 µg/cm^3^) was quantified.

Very high level of MDPBP in blood confirmed that high dose of this drug has been intaken before death. This may suggest overdose of this drug what probably finally contributed to victim’s death. The presence of small amount of cannabinoids’ metabolite proves early hashish or marijuana use with no significant effect of these toxic substances. Fatal cases caused by overdose of cannabinoids are very rare and often associated with taking other psychoactive substances at the same time. Moreover, there are very little data about the human pharmacokinetics and pharmacodynamics of MDPBP. Therefore, there is no information in the literature about interaction of synthetic cathinones and cannabinoids. It can be easily explained by the fact that these drugs are antagonists and interact with different receptors in human organism. Cannabinoids affect receptors CB_1_ and CB_2_ while cathinones, similar to amphetamines, inhibit monoamine uptake receptors and exert stimulant effect (increasing concentration of catecholamines). However, taking marijuana causes reduction of mental functioning what may lead to taking other drugs in uncontrolled doses [7, 11].

To our best knowledge, this is the first work that presents determination of MDPBP in blood by GC-EI-MS method and the third fatal accident confirmation of MDPBP abuse. The first fatal case was reported in the UK in 2011 and there was 1.55 µg/cm^3^ of MDPBP in victim’s blood determined [12]. The second one was also reported in Poland in 2014 and concerned a 19-year-old man [6]. Toxicological examination by LC–MS/MS technique revealed a concentration of MDPBP in blood 7.01 µg/cm^3^, which is comparable to the result obtained in our case (9.32 µg/cm^3^). Those authors also reported case of four drivers controlled by the Police, because of suspicions of driving under the influence of drugs. Analysis of blood samples collected from these drivers showed the concentration of MDPBP ranging from 22 to 92 ng/cm^3^. Above-mentioned, fatal concentrations of MDPBP were even hundred times higher than values determined in blood of drivers suspected of driving under the influence of drugs. Another confirmation case of MDPBP abuse, but non-fatal, was reported in Czech Republic in 2013 [13]. A 36-year-old man, who was treated for his long-term ethanol addiction and a habit of experimenting with new drugs available in the local black market shops, admitted to have taken an illegal powdered drug that was distributed under the name “Funky”, to overcome the symptoms of ethanol withdrawal. In his urine MDPBP and 3′-hydroxy-4′-methyl-α-pyrrolidinobutyrophenone as well as 4′-hydroxy-3′-methyl-α-pyrrolidinobutyrophenone, in view of common *m*/*z* value on mass spectra, was detected. These two compounds were recognized as main metabolites of MDPBP. Suggested metabolic pathway was similar to proposed schemes by Zaitsu et al. obtained by demethylenation followed by O-methylation [14].

## Conclusion

Although various human sample matrices are available in forensic toxicology analysis, blood is of the first choices being a reference biological material, and one of the most commonly used matrixes for drug determination with possibility of quantitative and qualitative interpretation. However, the determination of drugs in blood proves merely taking drugs without the possibility to estimate the route of administration (it is usually estimated based on the analysis of various biological materials). Due to the rapidly growing numbers of new psychoactive substances available on black market and the large variability of the drugs in structure and in concentration, the forensic investigation of intoxications or fatalities is relatively difficult. Therefore, new methods with fully validated procedure for the analysis of novel drugs are of the utmost importance.

Low limit of quantification, high recoveries, and good repeatability of results make proposed method for the determination of MDPBP in blood sufficient for identification both fatal and toxic cases with even small concentrations of MDPBP. It provides to be applicable in comprehensive forensic investigations.

## Experimental

Methanolic solution of 3,4-methylenedioxy-α-pyrrolidinobutiophenone hydrochloride (MDPBP·HCl) at concentration 1.0 mg/cm^3^ (as a free base) was purchased from Cayman Chemicals (Ann Arbor, MI, USA) and rac-methamphetamine-D_5_ (mAMP-D_5_) at concentration 0.1 mg/cm^3^ was obtained from LGC Standards (London, United Kingdom). Both solutions were used as standards.

All solvents used were of HPLC grade purity and were obtained from SIGMA ALDRICH (St. Louis, USA). Trifluoroacetic anhydride (TFAA) and 1-chlorobutane were ≥99 % purity (analytical grade) and were also purchased from SIGMA ALDRICH. Deionized water was purified with a Synergy 185 ultra-pure water system (Millipore, Milford, MA, USA). Hydrochloric acid at concentration 35–38 %, potassium carbonate (K_2_CO_3_), and sodium chloride (NaCl) were of analytical grade and were obtained from POCH (Gliwice, Poland). The 5 mol/dm^3^ K_2_CO_3_ and saturated NaCl solutions were prepared by dissolving above-mentioned salts in deionized water.

### Samples

Blood samples were obtained during autopsy and were stored at +4 °C before the analysis. For method validation purpose blood was obtained from the local blood bank (Gdańsk, Poland) from subjects without drug history. When analyte concentration in a sample was initially higher than the calibration curve range, the sample was diluted with drug-free blood, the extraction procedure was performed again and extract was re-injected.

### Preparation of standard and calibration solutions

Standard of MDPBP·HCl was diluted with methanol to make 25 µg/cm^3^ stock solution. Purchased mAMP-D_5_ standard solution was used as the internal standard (IS). mAMP-D_5_ was chosen as IS in view of that it does not occur naturally in the body fluids and that deuterated form of MDPBP was not commercially available. The calibration solutions were prepared by mixing appropriate amount of the stock solution with drug-free blood to obtain concentrations 25, 50, 100, 250, 500, 1000 ng/cm^3^. The added volume of IS standard solution at concentration 0.1 mg/cm^3^ to each sample was 5 mm^3^. Finally, the whole extraction procedure was performed.

### Sample preparation

Sample preparation was performed using modified method according to in-house prepared method for the determination of amphetamines and piperazines in blood and urine by liquid–liquid extraction procedure with derivatization followed by GC-EI-MS analysis. Blood (1 cm^3^), standard solution of IS (5 mm^3^), K_2_CO_3_ at concentration 5 mol/dm^3^ (2 cm^3^), saturated NaCl (2 cm^3^), and acetonitrile (2 cm^3^) were added to the screw-capped glass centrifuge tube. The tube was vortex-mixed for 1 min. Subsequently, 1-chlorobutane (2 cm^3^) was added and sample was vortex-mixed for 2 min. The solution was centrifuged for 3 min at 3000 rpm. The top layer was then transferred to a new glass tube. An additional 2 cm^3^ of 1-chlorobutane were added to the blood after first extraction, the tube was vortex-mixed for 2 min and then centrifuged for 3 min at speed 3000 rpm. The top layer was added to tube containing the first extracts. The bottom layer was discarded. Then 100 mm^3^ of hydrochloric acid solution in methanol (1:9, v/v) was added. Then, extracts were evaporated to dryness with an inert gas stream (nitrogen) at 40 °C and reconstituted in 50 mm^3^ ethyl acetate. Derivatization was performed with 50 mm^3^ TFAA (20 min, 55 °C). Finally, solution was evaporated to dryness and residue was dissolved in 50 mm^3^ of ethyl acetate. An aliquot of 2 mm^3^ volume was injected into the GC–MS system.

### Instrumentals

The chromatographic separation was carried out using Trace GC gas chromatograph equipped with autosampler AS 3000, split/splitless injector and mass spectrometry detector Trace DSQ (Thermo Finnigan). The analytes were separated on capillary column ZB-5 MS 30 m × 0.25 mm i.d., 0.25 µm film thickness (Phenomenex) with helium as a carrier gas (1.0 cm^3^/min). Split injection mode was used (10:1). The oven temperature was programmed as follows: 1 min at 50 °C, then 30 °C/min up to 160 °C, 10 °C/min ramp up to 250 °C, and finally 30 °C/min ramp to 300 °C. Post-run conditioning was set at 300 °C for 10 min in order to eliminate carry-over effect. Inlet and detector mass spectrometry (MS) source ions temperatures were 260 and 280 °C, respectively. For full-scan acquisition, the MS was operated in positive electron impact mode (electron energy 70 eV) and the mass detection range was *m*/*z* 30–380. The following ions were chosen for selected ion monitoring mode (SIM) for identification and quantification: MDPBP, *m*/*z* 70, 149, 112; IS, 158, 113, 119. The underlined ions were used for quantification. These ions were chosen on the basis of their abundance and that they were also among the most specific ions present.

### Validation procedure

Full validation is important for a new drug entity and for proper toxicological interpretation and establishing new references criteria. The method was validated following the accepted criteria for bioanalytical method validation [9, 10]. We evaluated selectivity, matrix effect, linearity, limit of detection (LOD), limit of quantification (LOQ), carry-over effect, trueness, recovery, and repeatability.

### Selectivity, calibration curve and matrix effect

Selectivity experiments were carried out with six blood samples obtained from various subjects to confirm that no substances were present in the retention times of analyte and internal standard. The whole extraction procedure was performed and extracts were injected to GC–MS system.

Six-point calibration curve (25, 50, 100, 250, 500, and 1000 ng/cm^3^) was constructed in triplicate by plotting the ratio of the MDPBP peak area to the peak area of the IS versus the MDPBP concentrations. Two calibration curves have been done according to Matuszewski et al. [15]: solutions of analyte were prepared in methanol as well as in extracts obtained from drug-free blood to determine matrix effect and decide which calibration approach should be applied. Matrix effect was calculated as follows (Eq. 1) [15]:

1 $$ME \;\left[ {\text{\% }} \right] = \frac{{a_{m} }}{{a_{s} }}{ \times }100 \;{\text{\% }}$$

where *a*_*m*_ is the slope in extracts spiked with analytes, and *a*_*s*_ is the slope in solvent.

### Linearity, limit of detection (LOD), limit of quantification (LOQ)

The linearity was investigated in concentration range 25–1000 ng/cm^3^. The weighing factor of 1/*x* was applied to calibration curve in order to increase the accuracy in the lower concentration range. The limit of detection (LOD) was established based on following formula: LOD = 3.3·*S*_*b*_*/a*, where *S*_*b*_ is the standard deviation of the intercept and *a* is the slope of the calibration curve. The limit of quantification (LOQ) was calculated as three times LOD value [16].

### Carry-over effect, trueness, recovery, and repeatability

The carry-over test was performed by injection to GC–MS system the highest concentration of analyte from the calibration curve followed by methanol solution. The test was performed six times. The trueness of the results was measured by recoveries using spiked blood as a matrix at three different concentration levels (low-25, medium-250, and high-1000 ng/cm^3^). Each sample was analyzed in sixfold on the same day (*n* = 6). Repeatability was assessed as inter-day precision by analyzing blank blood samples also spiked at the above-mentioned tree concentration levels (low, medium, and high). The analyses were repeated during the next three consecutive days. Inter-day repeatability was expressed as recovery and CV of between-days averages. Each sample was analyzed in sixfold each day. The results obtained by comparing the analyte to IS peak area ratio for the spiked and extracted blank blood samples with the corresponding extracts spiked with standard solutions of analyte (matrix-match calibration curve). In all cases, internal standard was spiked post-extraction to avoid its loss during extraction.
